# Looping Out Introns to Help Splicing

**DOI:** 10.1371/journal.pbio.0040041

**Published:** 2006-01-10

**Authors:** Richard Robinson

One of the most surprising discoveries in molecular biology was that a gene's coding region is broken up into smaller pieces (the exons) interrupted by noncoding portions called introns. After the DNA is transcribed into RNA, and before the RNA can leave the nucleus, the introns must be cut out and the exons spliced together.

Since introns were discovered in 1977, the details of the splicing operations have been a major object of study. For splicing to occur, the ends of an intron must be brought into close proximity, and a number of proteins have been identified that aid this process. However, the function of one group of these proteins, called the hnRNP proteins, which are known to associate with prespliced RNAs, has not been clear. To date, the most accepted role for a subgroup of these proteins, the hnRNP A/B proteins, has been a negative one, since binding of these proteins to certain exons can prevent their inclusion in the mature RNA. In this issue, Rebecca Martinez-Contreras, Benoit Chabot, and colleagues show that when hnRNP A/B or hnRNP F/H proteins bind to intron sequences near splicing signals, they can stimulate splicing.

The authors began by making long artificial RNA segments, which are poorly spliced due to the more than 1,000 nucleotides separating the two ends of their introns. By inserting hnRNP A/B–binding sites in the intron near the future splice junctions, they could increase splicing efficiency 4-fold. The binding sites did not have to be on the RNA itself, as long as they stayed close to the ends of the intron, as shown when the authors tethered short pieces of RNA to each end of the intron. These short RNAs contained the binding sites on their tails, which hung loose in approximately the right place next to the intron. This allowed the hnRNP A/B proteins to take up position near the ends of the introns, and splicing efficiency was increased. When binding sites were placed well into the interior of the intron, either on the intron itself or on an RNA tail, splicing was inefficient. Similar results were found when binding sites were inserted for a different binding protein group, hnRNP F/H. [Fig pbio-0040041-g001]

**Figure pbio-0040041-g001:**
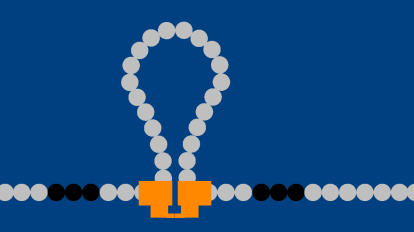
Two hnRNP proteins interact to connect the two ends of an intron, forming a loop to help the cell's splicing machinery remove the intron

The authors propose a model for these results in which the bound hnRNP proteins interact with one another, clasping the two ends of the intron together, forming a loop to help the splicing machinery remove the intron. The authors further support their model by showing that splicing could also be stimulated just by inserting complementary RNA sequences at each end of the intron. These have the ability to bind to one another, forming the intron into a loop.

However, they note that in some introns, only one hnRNP A/B site, positioned on the upstream end, is needed to promote splicing, and it does so nearly as well as when sites at both ends are present. The reason may lie in the particular introns that display this behavior—they contain a sequence which may itself bind an hnRNP A/B protein, thus providing the missing binding site and leading to loop formation. Confirmation or refutation of this hypothesis will have to await future experiments. Notwithstanding, the model is generally appealing because the two ends of many human introns are enriched in binding sites for these proteins. Overall, this mechanism suggests that hnRNP proteins can remodel the structure of prespliced RNAs, a property, the authors suggest, that could be important for both splicing and alternative splicing in a wide variety of genes.

